# Infrared Thermographic Assessment of Exercise-Induced Changes in Local Surface Temperature in Horses Following Treadmill Training: Preliminary Findings

**DOI:** 10.3390/ani16162517

**Published:** 2026-08-12

**Authors:** Cristian Zaha, Tudor Căsălean, Cristina Văduva, Alexandru Cireșan, Paula Nistor, Vlad Cocioba, Larisa Schuszler, Ciprian Rujescu, Janos Degi, Roxana Dascălu

**Affiliations:** 1 Horia Cernescu Research Unit, Faculty of Veterinary Medicine, University of Life Sciences “Regele Mihai I” from Romania, 300645 Timisoara, Romania; cristian.zaha@usvt.ro (C.Z.); tudor-mihai.casalean.fmv@usvt.ro (T.C.); cristina.vaduva@usvt.ro (C.V.); alexandru.ciresan@usvt.ro (A.C.); paula.nistor@usvt.ro (P.N.); vlad-mihai.cocioba.fmv@usvt.ro (V.C.); larisaschuszler@usvt.ro (L.S.); janosdegi@usvt.ro (J.D.); 2 Management and Rural Development Department, Faculty of Management and Rural Tourism, University of Life Sciences “Regele Mihai I” from Romania, 300645 Timisoara, Romania; rujescu@usvt.ro

**Keywords:** treadmill exercise, thermography, local temperature changes, horses

## Abstract

Background: Physical exercise induces physiological adaptations associated with increased muscular activity, metabolic heat production, and changes in body surface temperature. Infrared thermography has emerged as a non-invasive technique for assessing these thermal responses in horses. The present study aimed to evaluate the ability of infrared thermography to detect changes in mean local surface temperature and to qualitatively assess alterations in thermal distribution patterns following treadmill exercise. Methods: Three clinically healthy Warmblood horses were monitored over 10 consecutive training sessions. Thermographic examinations were performed before exercise (T1) and immediately after exercise (T2) in two anatomical regions: Bx1, corresponding to the deltoid musculature, and El1, corresponding to the gluteal musculature. Mean local surface temperatures were analyzed using linear mixed-effects models. Results: Exercise resulted in significant increases in mean local surface temperature in both evaluated regions. In the Bx1 region, mean temperature increased from 33.75 °C before exercise to 35.36 °C after exercise (F = 968.32, *p* < 0.001), whereas in the El1 region it increased from 32.37 °C to 33.33 °C (F = 296.31, *p* < 0.001). Qualitative evaluation of thermographic images revealed consistent alterations in thermal distribution patterns, characterized by a greater extent of high-temperature areas following exercise. Conclusions: Infrared thermography proved to be an effective method for detecting exercise-induced changes in local surface temperature and may serve as a valuable tool for monitoring training-related physiological adaptations in horses.

## Simple Summary

Regular physical exercise induces physiological changes that increase muscle activity and body surface temperature in horses. Monitoring these thermal responses may provide useful information about training adaptation and musculoskeletal function. In this study, infrared thermography was used to evaluate temperature changes in two major muscle regions before and after treadmill exercise in clinically healthy horses. Exercise produced clear increases in local surface temperature together with visible changes in thermal distribution patterns. These findings suggest that infrared thermography is a practical, non-invasive technique for monitoring exercise-induced thermal responses and may support training management and health assessment in equine athletes.

## 1. Introduction

Physical training is a fundamental component of equine management and athletic preparation, contributing to the development of cardiovascular fitness, musculoskeletal strength, and overall performance capacity [1,2]. Regular exercise induces a series of physiological adaptations that enhance the horse’s ability to perform work efficiently while reducing the risk of fatigue and injury. Monitoring the physiological responses associated with exercise is therefore essential for evaluating training effectiveness and optimizing conditioning programs [3,4].

During exercise, skeletal muscle contraction substantially increases metabolic heat production. To maintain thermal homeostasis, horses activate thermoregulatory mechanisms involving enhanced peripheral blood flow and heat dissipation through the skin [5,6,7]. These thermal responses are closely related to local perfusion and tissue metabolism, making body surface temperature a useful indicator of physiological adaptation to exercise [8,9].

The assessment of body surface temperature has gained increasing attention in equine sports medicine as a means of evaluating exercise-induced physiological changes [7,10]. Traditional methods of temperature assessment are often limited to measurements of core body temperature and provide little information regarding localized thermal responses [10,11]. In contrast, infrared thermography offers a non-invasive, non-contact imaging technique capable of detecting and quantifying surface temperature distribution in real time [12,13]. The technique is based on the measurement of infrared radiation naturally emitted by body tissues, generating thermal images that reflect underlying physiological processes.

Infrared thermography has become a valuable tool in equine veterinary medicine for the assessment of inflammatory processes, musculoskeletal disorders, and circulatory abnormalities [14,15,16]. Because alterations in local surface temperature may precede the appearance of clinical signs, thermography is increasingly used not only for diagnostic purposes but also for monitoring equine health and performance [17]. While mean local surface temperature reflects the overall thermal response of a region of interest (ROI), maximum local surface temperature (peak temperature) may provide greater sensitivity to focal thermal changes associated with increased local metabolic activity and exercise-related muscular workload [18,19]. In addition, previous research in exercising horses has demonstrated significant relationships between infrared thermographic surface temperatures and blood lactate concentrations, suggesting that thermographic changes may reflect physiological responses associated with exercise workload [20]. Consequently, the combined evaluation of mean and maximum temperatures may provide complementary information regarding tissue responses to exercise [12,21]. Furthermore, several studies have reported significant post-exercise increases in body surface temperature, particularly in highly vascularized and muscular regions such as the neck, shoulder, elbow, and gluteal areas, suggesting that thermographic assessment may provide useful information regarding muscular activity and physiological responses to exercise [12,22,23].

Treadmill exercise represents a valuable experimental approach for investigating exercise physiology in horses [24]. Compared with field training, treadmill-based exercise allows greater control over speed, workload, duration, and environmental conditions, thereby reducing sources of variability and improving the standardization of experimental protocols [25,26]. Consequently, treadmill training provides an appropriate model for studying thermographic responses to physical effort and evaluating the usefulness of infrared thermography as a monitoring tool in equine athletes [27].

Therefore, the present study aimed to evaluate the ability of infrared thermography to detect exercise-induced changes in mean local surface temperature in horses. Particular attention was focused on the Bx1 and El1 regions, corresponding to the deltoid and gluteal musculature, respectively, in order to assess thermal responses associated with muscular activity before and after treadmill exercise. In addition, thermographic images were qualitatively examined for potential alterations in thermal patterns following exercise.

The null hypothesis was that no significant differences in mean local surface temperature would be observed between pre-exercise and post-exercise thermographic evaluations of the Bx1 and El1 regions.

## 2. Materials and Methods

### 2.1. Animals

The study was conducted on a group of three Warmblood horses with comparable age and morphological characteristics, all clinically healthy. Throughout the experimental period, the animals were housed individually under standard stable conditions.

Their diet consisted of lucerne hay provided in three daily portions, while water was available ad libitum. The inclusion criteria required the absence of lameness within the previous three months, normal findings on orthopedic examination, and no administration of anti-inflammatory medication in the month prior to the study.

A comprehensive clinical and orthopedic assessment was performed by an experienced equine veterinarian before the beginning of the study and was repeated during the experimental period, confirming the health status of all horses.

The experimental protocol included a total of 10 training sessions conducted on separate days under similar working conditions. Rectal temperature measurements obtained before exercise remained within the physiological range (37.0–37.8 °C), whereas following treadmill exercise an increase was observed, with values ranging from 38.6 °C to 39.4 °C.

### 2.2. Training Protocol and Thermographic Assessment

Local surface temperature was evaluated in selected muscular regions in three clinically healthy horses over the course of 10 training sessions performed on separate days. The experimental design focused on assessing thermal variations associated with physical exercise, by comparing measurements obtained before and after training.

Prior to the beginning of data collection, the horses underwent a short habituation period to both the training routine and the thermographic recording procedure, in order to reduce stress-related variability. All handling procedures, including exercise and image acquisition, were performed by the same operator to ensure consistency.

The exercise protocol consisted of a standardized training session carried out under similar conditions for all horses. The exercise sessions were performed using a Haiko 5000 treadmill (Loimaa, Finland). Before the beginning of the experimental period, the horses underwent a five-day acclimatization period to the exercise protocol. The same operator was responsible for both treadmill handling and thermographic image acquisition throughout the study. Each exercise session started with a 5-min walking phase at 1.2 m/s, followed by an increase in speed to 2 m/s for an additional 5 min. Subsequently, each horse performed a 20-min trotting phase at 4 m/s, after which the session concluded with 5 min of walking at 1.25 m/s (Video S1) [28,29]. Thermographic assessments of the Bx1 and El1 areas were carried out immediately after each exercise session. All procedures were conducted in a standardized manner during the entire ten-day experimental period.

**Figure 1:**
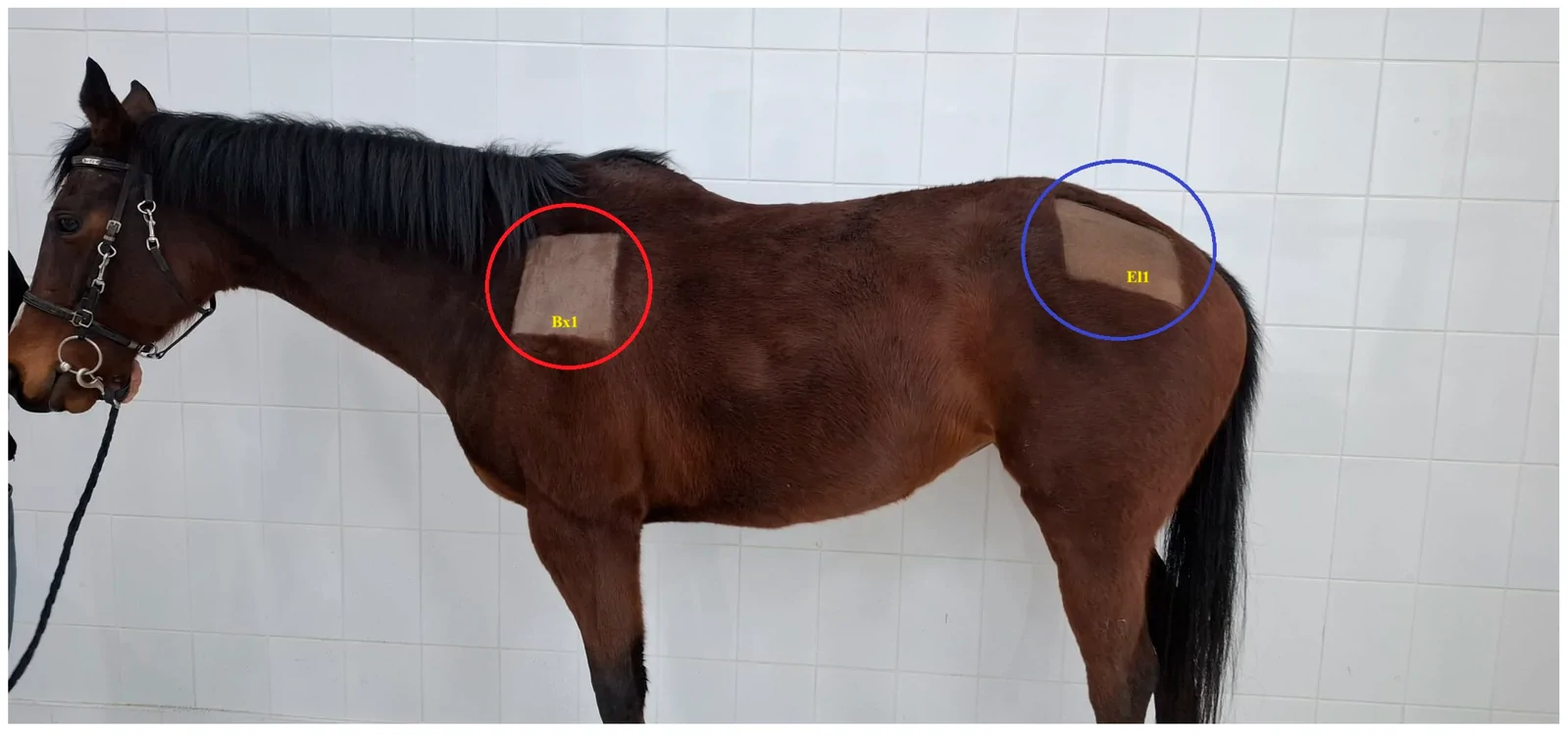
Lateral view of a horse showing the clipped anatomical regions selected for thermographic evaluation. The red rectangle indicates the Bx1 region, corresponding to the deltoid musculature, whereas the blue rectangle indicates the El1 region, corresponding to the gluteal musculature.

Thermographic assessments were performed at two different time points: before exercise (T1) and immediately after completion of the training session (T2). The regions selected for analysis included Bx1, corresponding to the deltoid musculature, and El1, representing the gluteal muscle region, both chosen due to their major involvement in equine locomotion and expected response to physical effort (Figure 1).

All evaluations were carried out indoors to minimize the influence of environmental factors on skin surface temperature. The hair over the BX1 and EL1 regions was clipped to approximately 0.8 mm before the first thermographic evaluation and re-clipped before each subsequent measurement session to maintain a uniform hair length throughout the study. Before each thermographic examination, horses were acclimatized for 30 min in the treadmill training room, where all thermographic images were acquired before and after exercise. During transfer from the stable to the training room, the horses were not exposed to direct sunlight, minimizing the influence of environmental factors on surface temperature measurements.

Thermal images were obtained using an infrared camera Flir E50 (FLIR Systems Inc., Wilsonville, OR, USA) positioned approximately 1 m from the animal and oriented perpendicular (90°) to the evaluated regions. The thermographic camera was operated using an emissivity setting of 0.95 and an image resolution of 240 × 180 pixels. The temperature range was set from −20 °C to 650 °C, with a thermal sensitivity of ≤0.05 °C. To reduce inter-observer variability, all recordings were performed by the same operator under standardized camera settings, including emissivity and temperature range.

Throughout the study, all three horses underwent their daily training sessions and thermographic evaluations on the same day between 8:00 a.m. and 2:00 p.m. Environmental conditions were maintained at an ambient temperature of 21–22 °C, a relative humidity of 70–75%, and no detectable air currents. Ambient temperature, relative humidity, and air movement were monitored using a Testo 435 instrument (Testo, Titisee-Neustadt, Germany).

Following image acquisition, thermographic images were processed and analyzed using FLIR Tools 5.X software by the same non-blinded operator. For all thermographic evaluations, the center of each clipped rectangular area, corresponding to the intersection of its diagonals, was used as the reference point for ROI placement to ensure consistent positioning throughout the study. Regions of interest corresponding to the BX1 and EL1 anatomical areas were then defined, and mean surface temperatures were extracted to assess exercise-induced thermal responses by comparing measurements obtained before and after training.

### 2.3. Statistical Evaluation

Three horses were monitored over 10 consecutive days, during which local surface temperatures were recorded in two anatomical regions (BX1 and EL1) before (T1) and after training (T2). Statistical analyses of both mean and maximum local surface temperatures were performed using linear mixed-effects models implemented in SAS OnDemand for Academics (SAS Studio 3.8). Mean and maximum local surface temperatures were considered dependent variables. Evaluation moment (T1 vs. T2) was included as a fixed effect, whereas horse was treated as a random effect to account for inter-individual variability. Because each horse was evaluated repeatedly on each study day, repeated measurements were modeled with evaluation moment specified as the repeated factor and the Horse × Day combination defined as the subject effect. This approach accounted for the correlation between paired pre-training and post-training measurements obtained from the same horse on the same day.

Data normality was assessed using the Shapiro–Wilk test. Paired-samples *t*-tests were performed to compare maximum local surface temperatures and rectal temperature before and after training. Linear regression analyses were used to investigate the relationship between rectal temperature (core body temperature) and maximum local surface temperature in the BX1 and EL1 regions before and after training. Differences between the regression slopes obtained before and after training were subsequently evaluated. Statistical significance was established at *p* < 0.05.

## 3. Results

### 3.1. Environmental Conditions During the Study

Environmental conditions remained stable throughout the study period. Daily measurements of ambient temperature and relative humidity are presented in Table 1. No detectable air currents were recorded during any thermographic evaluation session.

### 3.2. Animal Population

Rectal temperature measurements recorded before (T1) and immediately after (T2) treadmill exercise in the three horses throughout the study are presented in Table 2. Mild sweating was occasionally observed after exercise, particularly in the cervical and shoulder regions.

### 3.3. Thermography Scanning

#### 3.3.1. Bx1 Area

**Figure 2:**
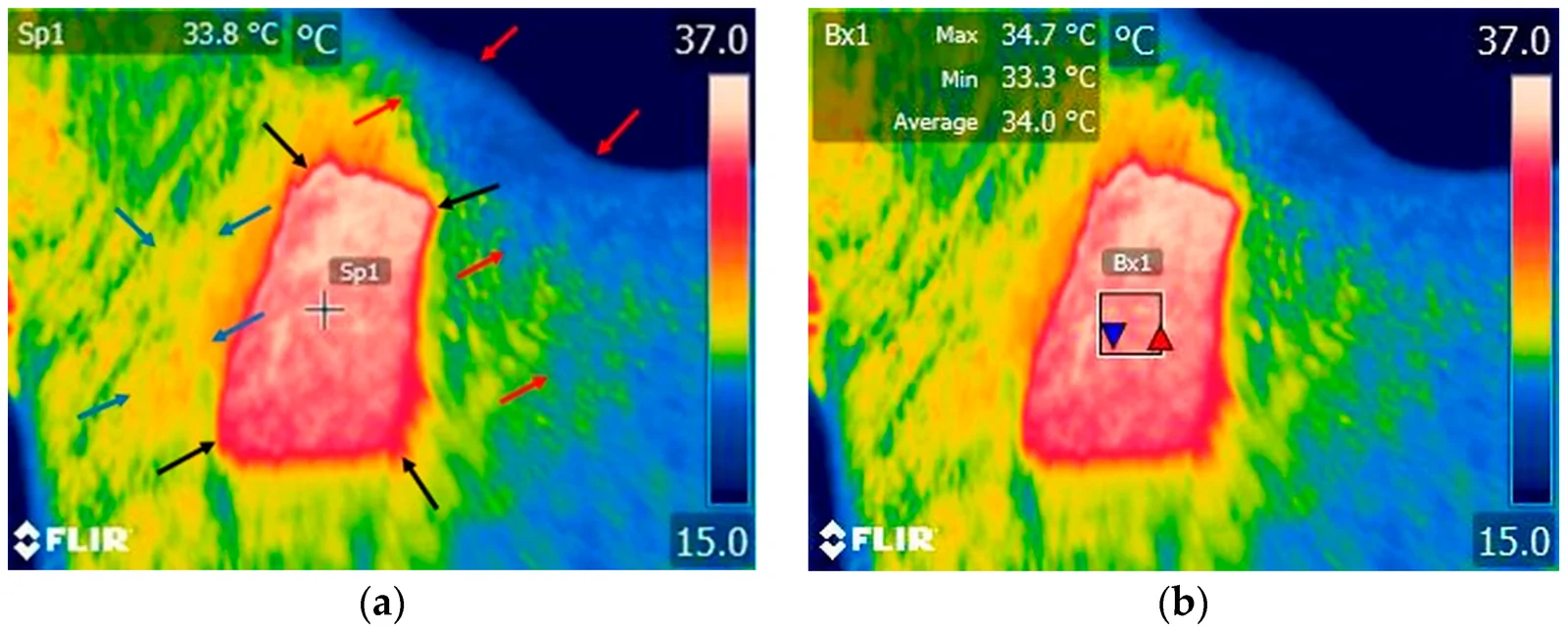
Thermographic image of the thoracal area before training: (**a**) Scanning image without Flir Tools software analysis: red arrows—variation area of low temperature, blue arrows—variation area of moderate temperature, black arrows—clipped hair area; (**b**) Scanning image with FLIR Tools software analysis: red triangle spot—maximum temperature recorded in the interest area and blue triangle spot—minimum temperature recorded in the interest area.

Thermographic images obtained prior to the training sessions revealed a thermal pattern predominantly characterized by areas of lower temperature, while the regions of interest displayed a heterogeneous temperature distribution represented by alternating red and white color patterns (Figure 2).

**Figure 3:**
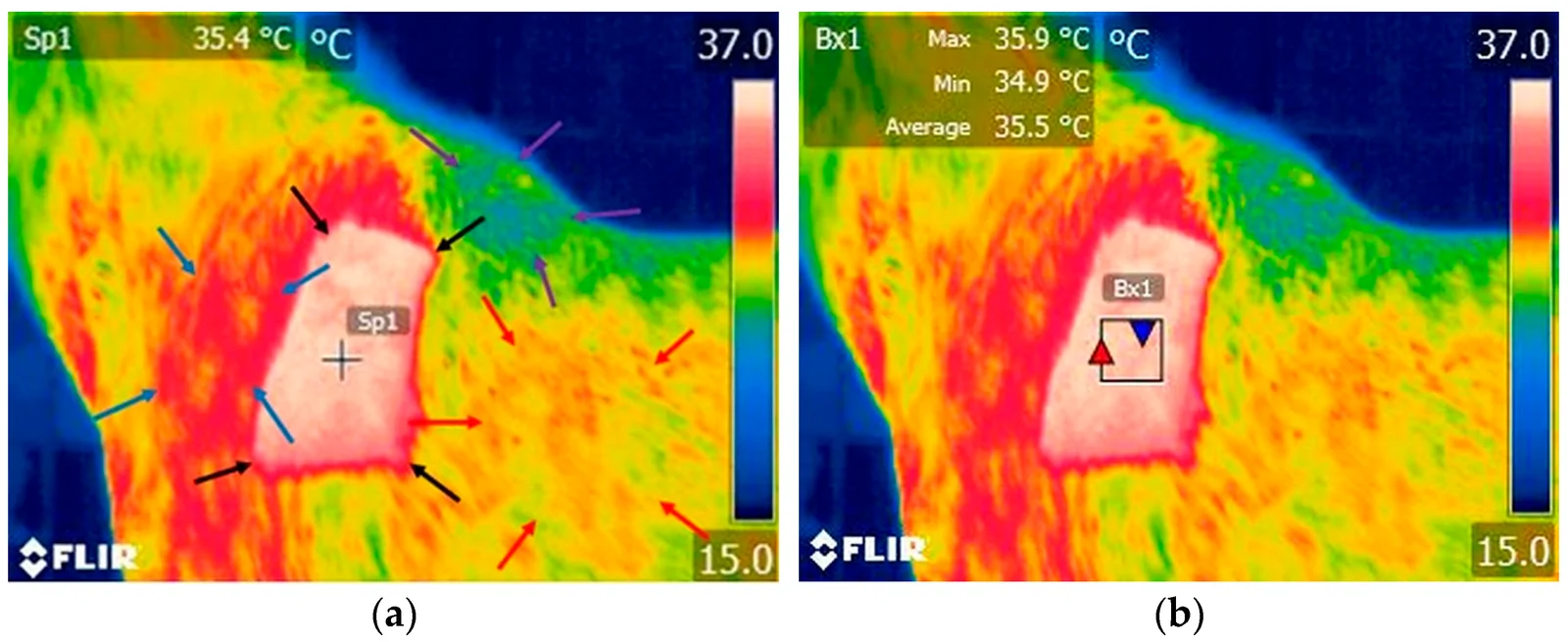
Thermographic image of the thoracal area after training: (**a**) Scanning image without Flir Tools software analysis: red arrows—variation area of moderate temperature, blue arrows—variation area of increased temperature, black arrows—clipped hair area with increased area of temperature; (**b**) Scanning image with FLIR Tools software analysis: red triangle spot—maximum temperature recorded in the interest area and blue triangle spot—minimum temperature recorded in the interest area.

Following exercise, noticeable modifications in the thermographic pattern were observed, characterized by the appearance of areas with increased temperature in the prescapular region. Within the regions of interest, the predominance of white coloration on the thermal images indicated higher surface temperatures compared to the adjacent anatomical areas (Figure 3).

#### 3.3.2. El1 Area

**Figure 4:**
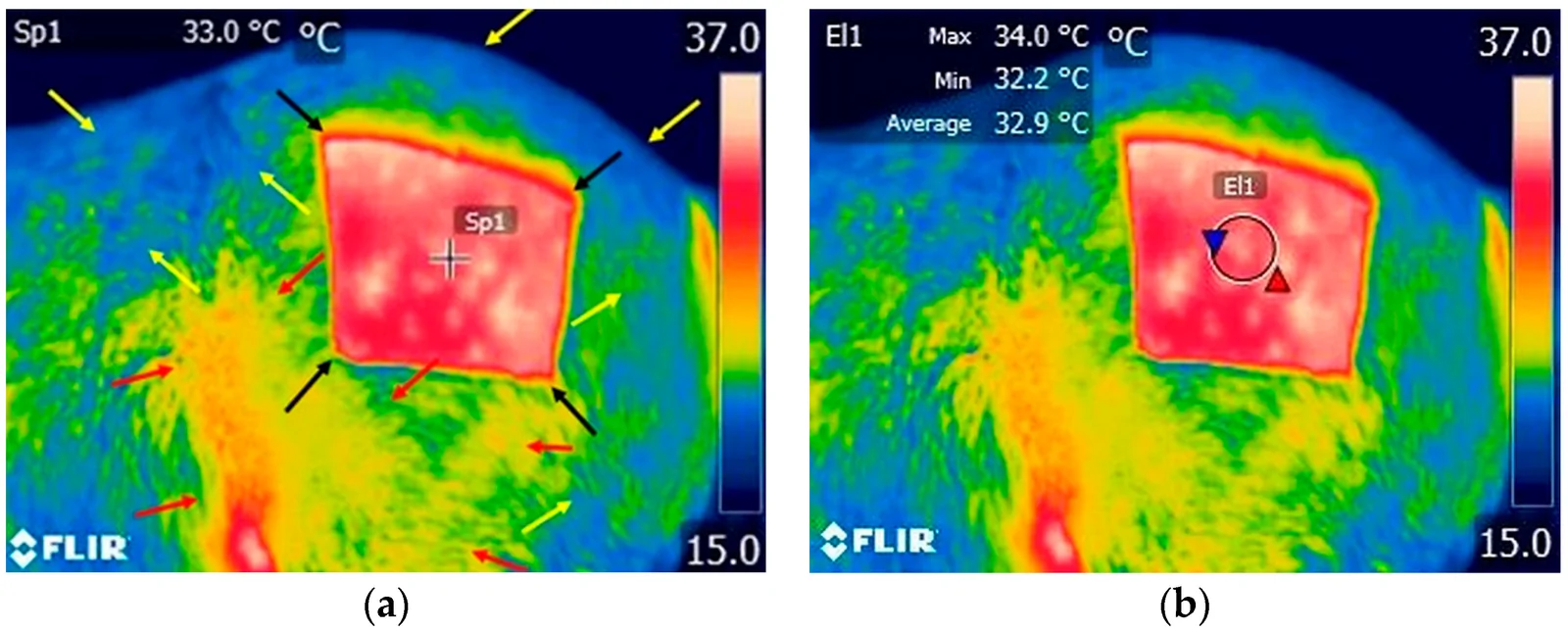
Thermographic image of the gluteal area before training: (**a**) Scanning image without Flir Tools software analysis: yellow arrows—variation area of cold temperature, red arrows—variation area of moderate temperature, black arrows—clipped hair area (**b**) Scanning image with FLIR Tools software analysis: red triangle spot—maximum temperature recorded in the interest area and blue triangle spot—minimum temperature recorded in the interest area.

Thermographic images recorded prior to exercise demonstrated a thermal pattern characterized predominantly by lower temperatures, represented by blue coloration, while within the region of interest a predominance of red coloration was observed, indicating moderate surface temperatures (Figure 4).

**Figure 5:**
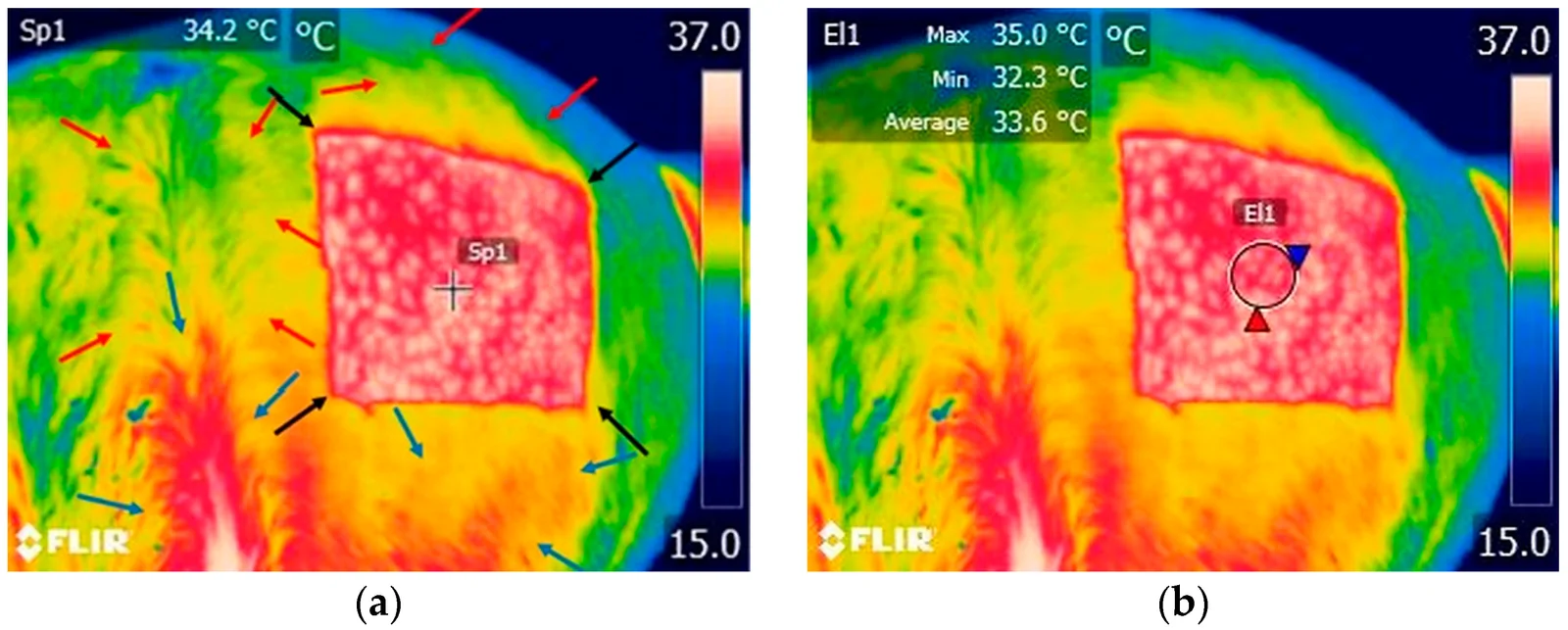
Thermographic image of the gluteal area after training: (**a**) Scanning image without Flir Tools software analysis: red arrows—variation area of moderate temperature, blue arrows—variation area of increased temperature, black arrows—clipped hair area (**b**) Scanning image with FLIR Tools software analysis: red triangle spot—maximum temperature recorded in the interest area and blue triangle spot—minimum temperature recorded in the interest area.

Following the training session, an increase in local muscular temperature was observed in the gluteal region, accompanied by a marked elevation in temperature within the region of interest, where white coloration became predominant on the thermographic images (Figure 5).

### 3.4. Group Comparison

#### 3.4.1. Evaluation of Temperature Differences in the Bx1 Region

The use of a Mixed Linear Model demonstrated significant differences between the temperatures recorded before and after training in the Bx1 region, with a test statistic of F = 968.32, *p* < 0.001, and DF = 56. The mean local surface temperature increased significantly from 33.75 °C before exercise to 35.36 °C after training, while the maximum local surface temperature increased from 34.20 °C to 35.80 °C (Table 3, Table S1).

**Figure 6:**
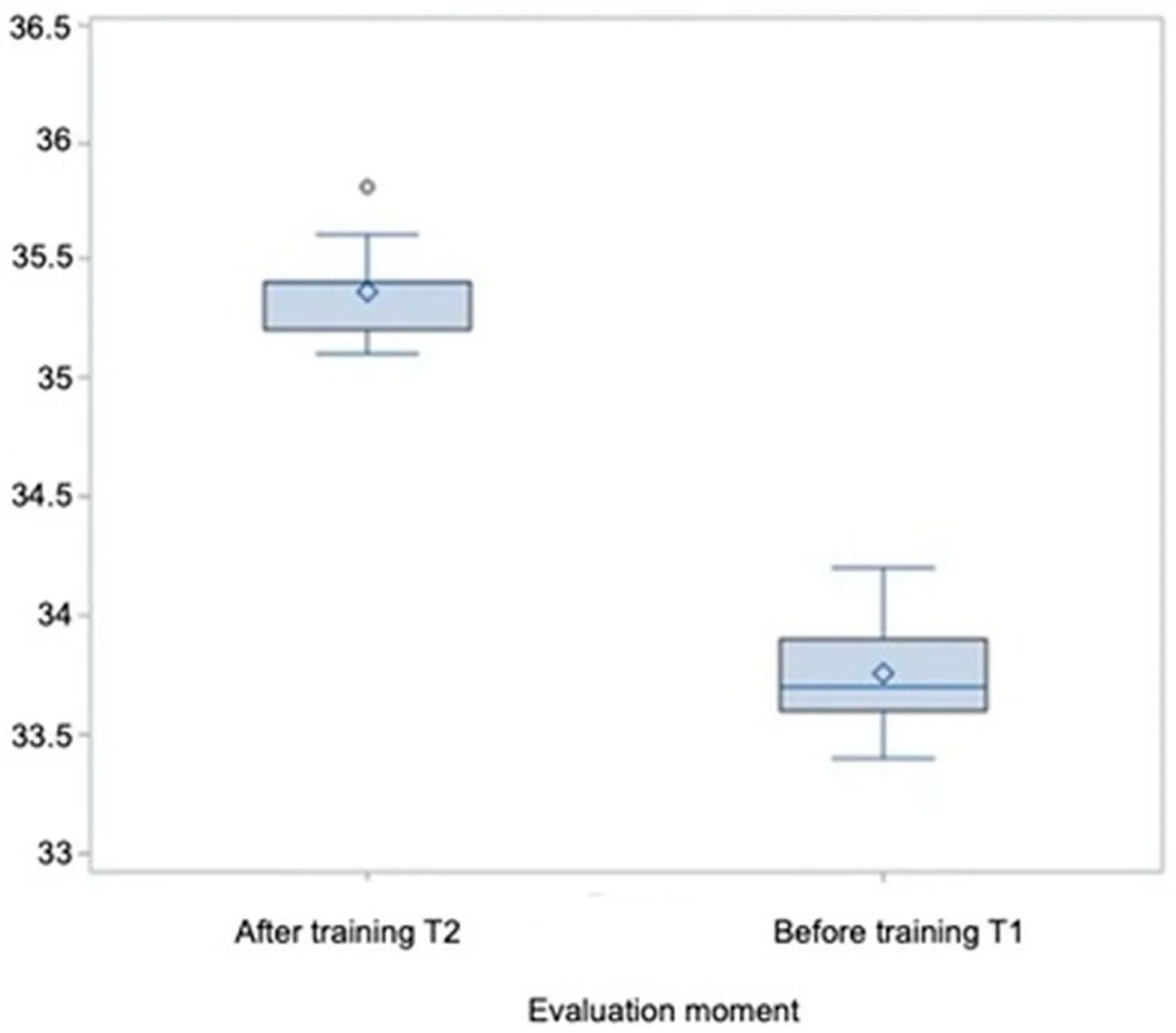
Boxplot comparing mean local surface temperature values recorded in the BX1 region before (T1) and immediately after (T2) treadmill exercise in all horses throughout the study period, ◦—outliers.

**Figure 7:**
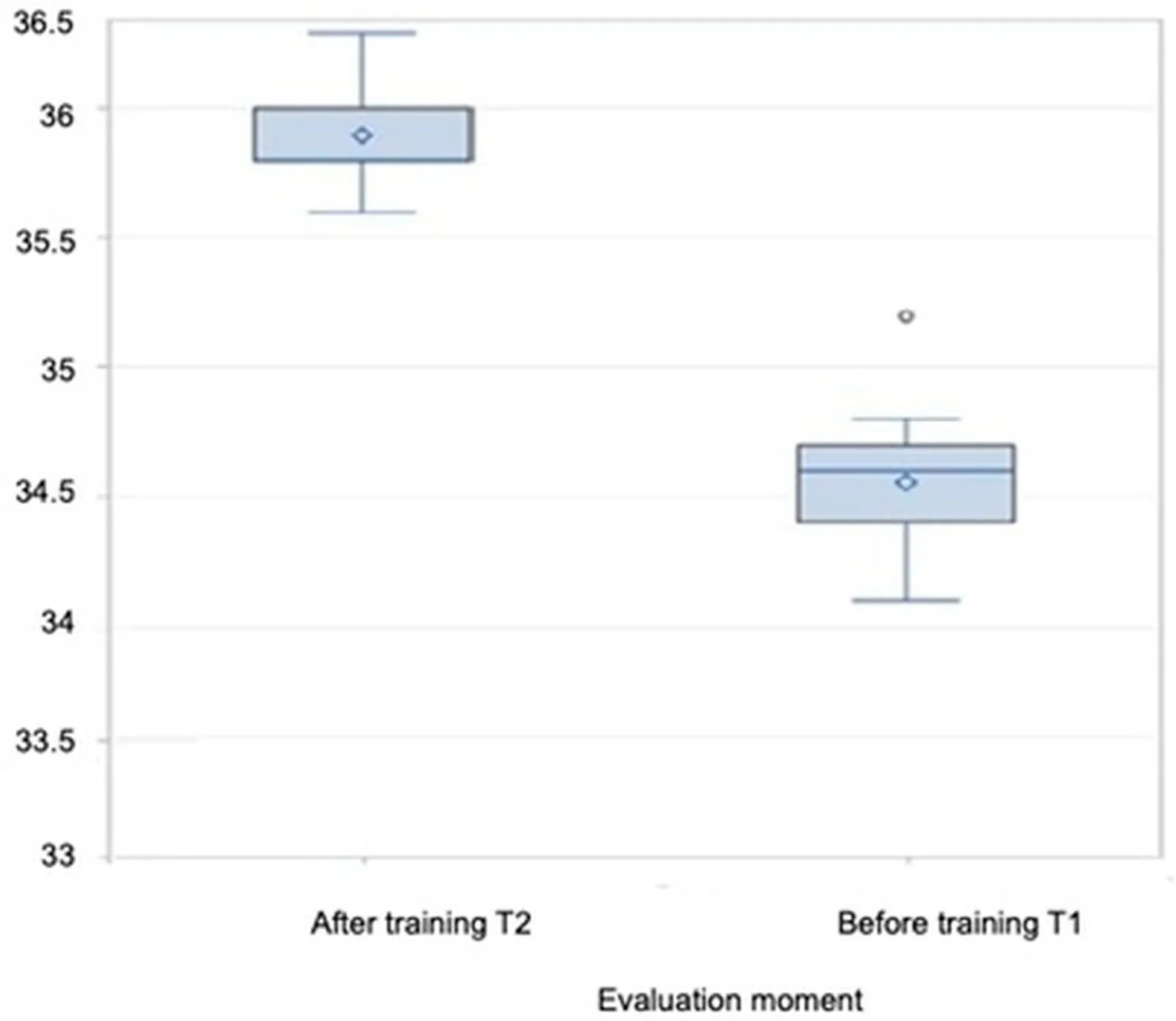
Boxplot illustrating the distribution of maximum local surface temperature values recorded in the BX1 region before (T1) and immediately after (T2) treadmill exercise in all horses throughout the study period, ◦—outliers.

The boxplots presented in Figure 6 and Figure 7 illustrate the distributions of mean and maximum local surface temperature values, respectively, recorded in the BX1 region before (T1) and immediately after (T2) treadmill exercise.

#### 3.4.2. Evaluation of Temperature Differences in the El1 Region

Similarly, in the second evaluated body region, El1, statistical analysis revealed significant differences between the temperatures recorded before and after training. The Mixed Linear Model analysis yielded values of F = 296.31, *p* < 0.001, and DF = 56. The mean local surface temperature increased from 32.37 °C before exercise to 33.33 °C after training, while the maximum local surface temperature increased from 32.80 °C to 33.80 °C (Table 4 and Table S2).

**Figure 8:**
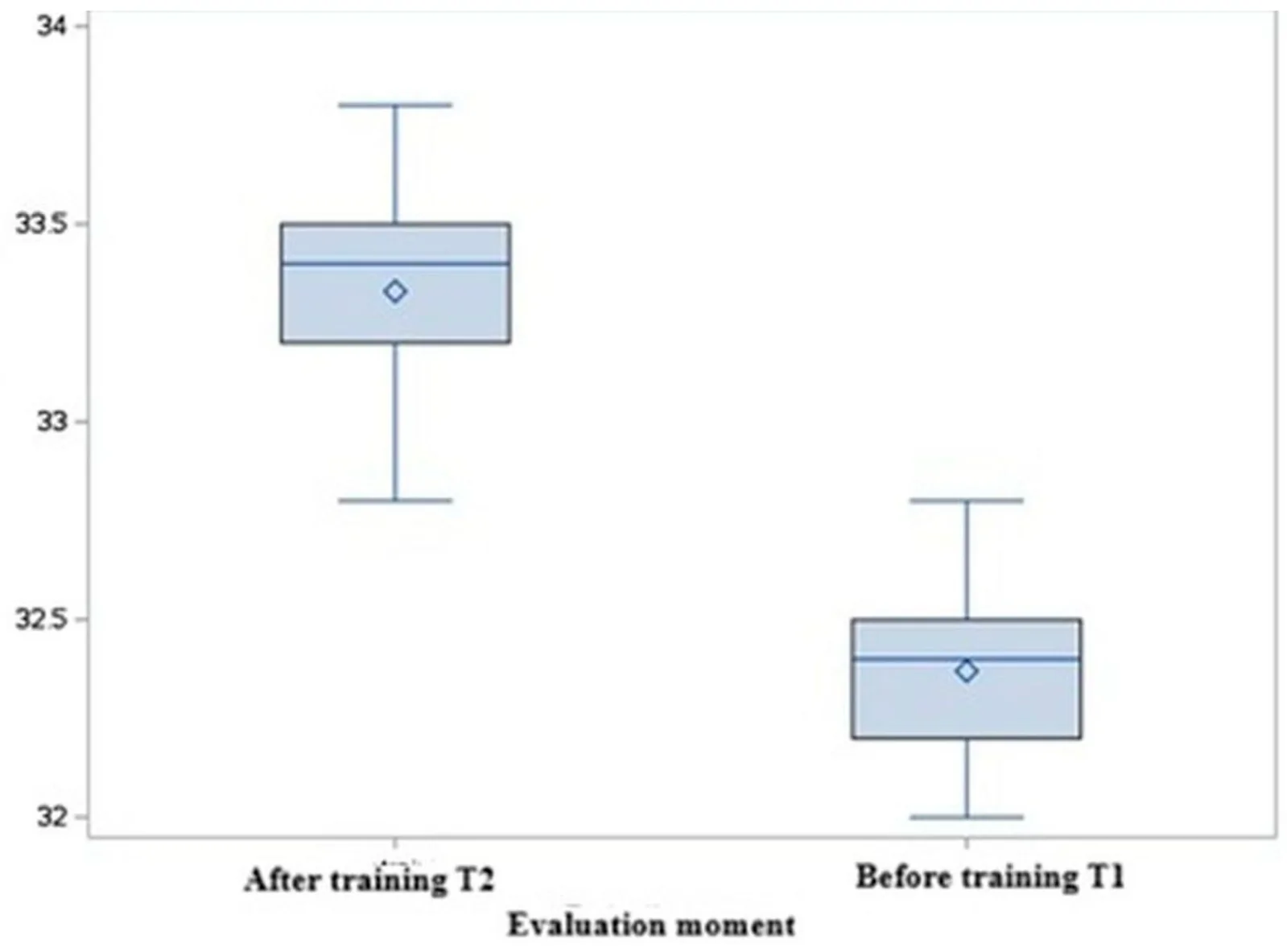
Boxplot illustrating the distribution of mean local surface temperature values recorded in the EL1 region before (T1) and immediately after (T2) treadmill exercise in all horses throughout the study period.

**Figure 9:**
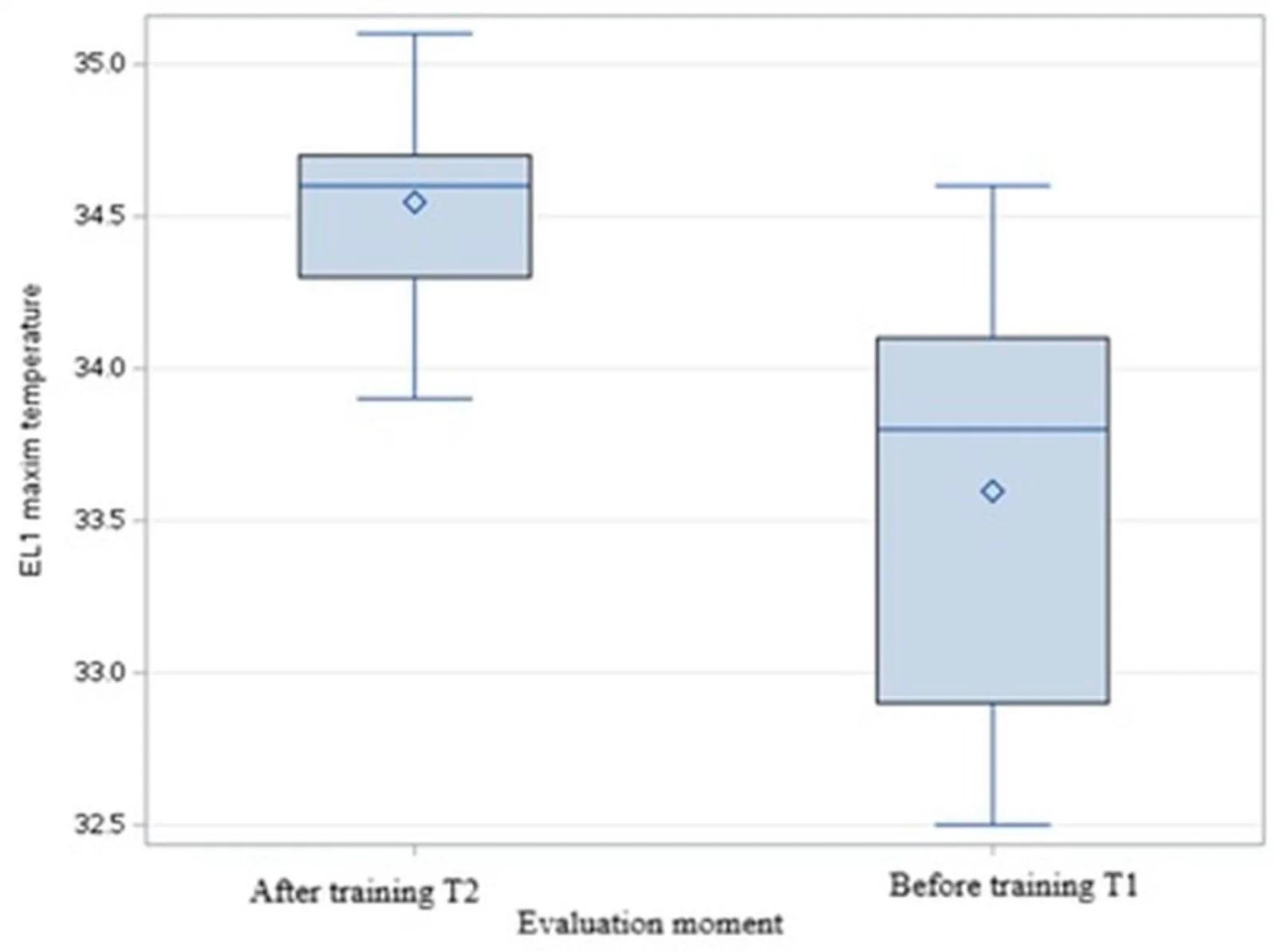
Boxplot illustrating the distribution of maximum local surface temperature values recorded in the EL1 region before (T1) and immediately after (T2) treadmill exercise in all horses throughout the study period.

The boxplots presented in Figure 8 and Figure 9 illustrate the distributions of mean and maximum local surface temperature values, respectively, recorded in the El1 region before (T1) and immediately after (T2) treadmill exercise.

#### 3.4.3. Regression Analysis of the Relationship Between Maximum Local Surface Temperature and Rectal Temperature for Bx1 Area

Linear regression analysis demonstrated the relationship between maximum local surface temperature and rectal temperature in the BX1 and EL1 regions before (T1) and immediately after (T2) treadmill exercise. Each regression model included 30 observations, corresponding to all measurements obtained from the three horses throughout the 10-day study period.

For the BX1 region, the regression equation before training was:

y = 43.6809 − 0.2431xy = 43.6809 − 0.2431xy = 43.6809 − 0.2431x

whereas after training it was:

y = 30.4148 + 0.1420xy = 30.4148 + 0.1420xy = 30.4148 + 0.1420x

**Figure 10:**
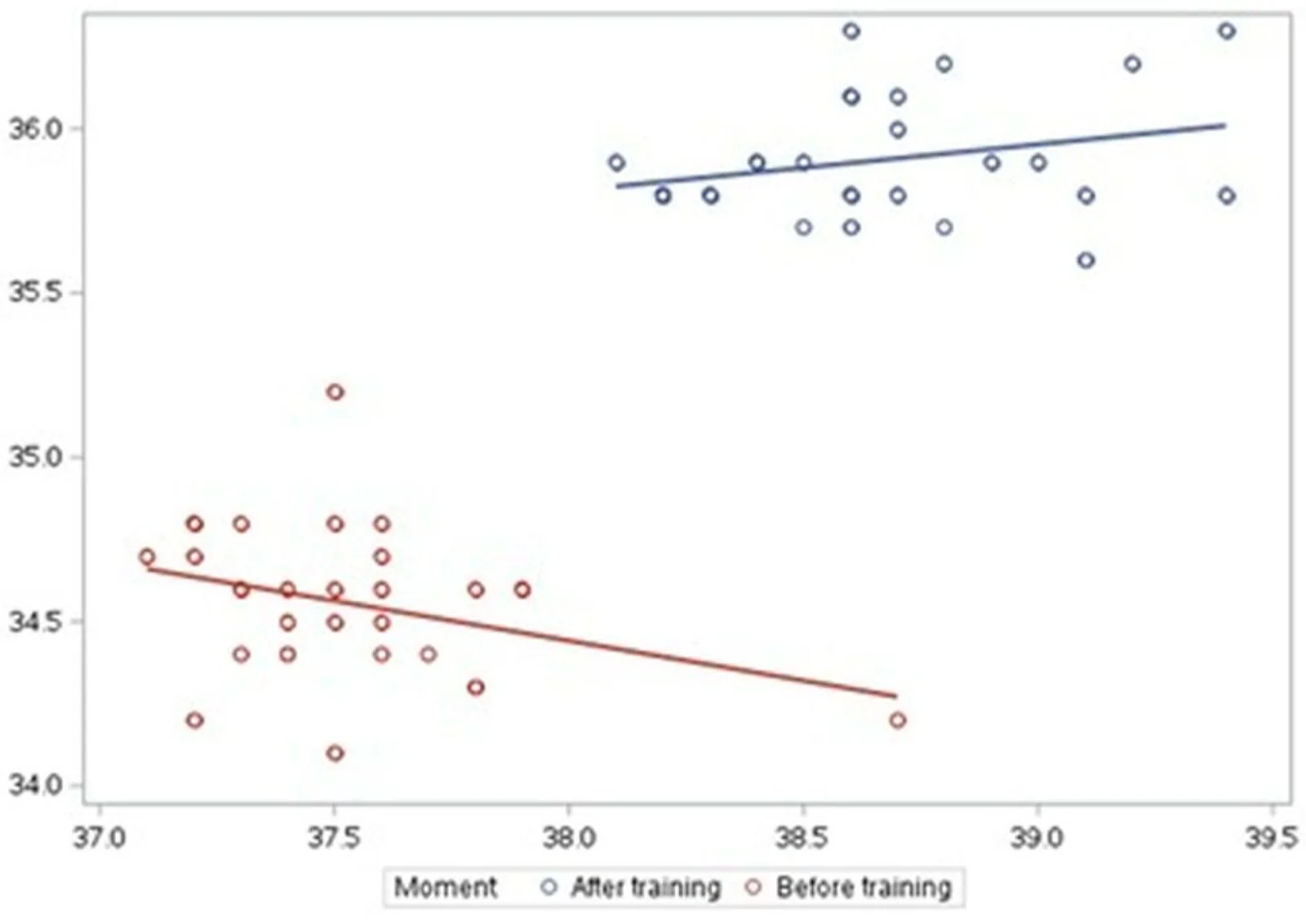
Regression plots illustrating the relationship between maximum local surface temperature in the BX1 region and rectal temperature, before (T1) and immediately after (T2) treadmill exercise.

The corresponding regression plots are shown in Figure 10.

Before training, the regression slope was negative (−0.2431), whereas after training it became positive (0.1420). The difference between the two slopes was statistically significant (*p* = 0.0172), indicating that the relationship between rectal temperature and maximum local surface temperature changed following treadmill exercise. Before training, horses with higher rectal temperatures tended to present lower maximum local surface temperatures in the BX1 region, whereas after training higher rectal temperatures were associated with higher maximum local surface temperatures.

For the EL1 region, the regression equation before training was:

y = 30.0996 + 0.0931xy = 30.0996 + 0.0931xy = 30.0996 + 0.0931x

whereas after training it was:

y = 33.3480 + 0.0310xy = 33.3480 + 0.0310xy = 33.3480 + 0.0310x

**Figure 11:**
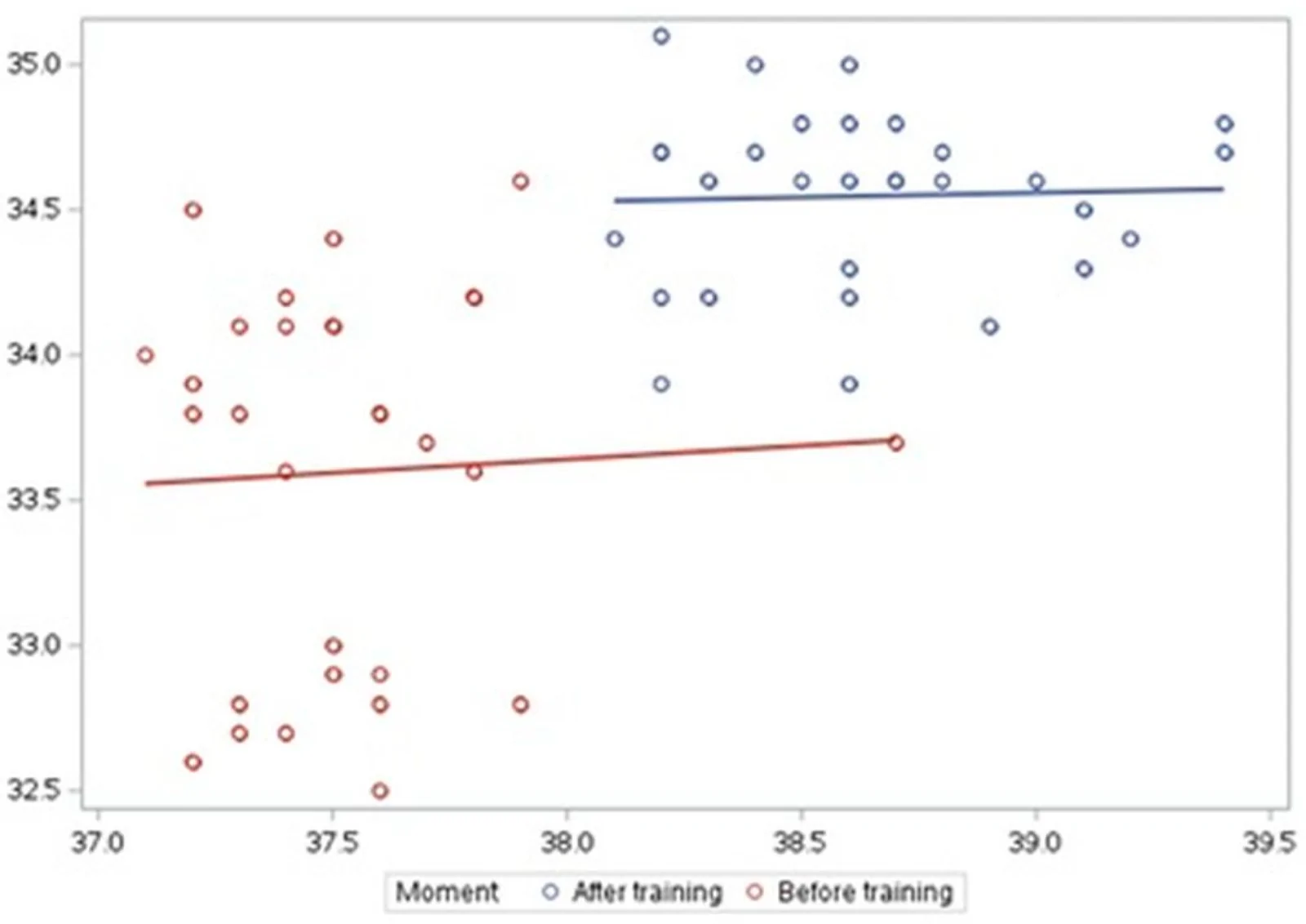
Regression plots illustrating the relationship between maximum local surface temperature in the El1 region and rectal temperature, before (T1) and immediately after (T2) treadmill exercise.

The corresponding regression plots are presented in Figure 11.

For the EL1 region, the regression slopes obtained before and after training did not differ significantly (*p* = 0.877), indicating that the relationship between rectal temperature and maximum local surface temperature remained unchanged following treadmill exercise.

The maximum local surface temperature datasets were normally distributed according to the Shapiro–Wilk test. No significant deviations from normality were detected for the BX1 region (*p* = 0.071), the EL1 region (*p* = 0.332), or rectal temperature (*p* = 0.975). Paired-samples *t*-tests demonstrated significant differences between measurements obtained before and immediately after treadmill exercise for all three variables (*p* < 0.0001). Specifically, maximum local surface temperatures in both the BX1 and EL1 regions were significantly higher after exercise than before exercise. Rectal temperature also increased significantly following treadmill exercise. A descriptive statistical summary of these variables is presented in Table 5.

## 4. Discussion

The results obtained in the present study do not support the null hypothesis that no significant differences in local surface temperature would occur between thermographic assessments performed before and after training. Furthermore, thermographic evaluation revealed evident modifications in the thermal distribution patterns observed within the investigated muscular regions.

Following exercise, both Bx1 and El1 regions demonstrated increased local surface temperatures, together with visible alterations in thermal distribution, characterized by the predominance of higher-temperature areas on the thermographic images. The increase in local surface temperature observed in horses after training can be explained by the physiological response of skeletal musculature to exercise [30,31]. During physical activity, the active muscle groups undergo an increase in metabolic activity and local blood perfusion, resulting in enhanced heat production [7,10]. Consequently, the heat generated within the muscles is transferred toward the skin surface, leading to higher thermographic values in the evaluated regions [31].

In addition to the increase in temperature values, the thermal pattern changed visibly following exercise. Before training, thermographic images were predominantly characterized by blue and yellow color distributions, indicative of lower surface temperatures, whereas after exercise the Bx1 and El1 regions displayed predominantly red and white areas, corresponding to increased local temperatures. Similar modifications in thermal patterns following exercise performed on a treadmill have also been reported by other authors, who observed changes in thermal distribution associated with increased muscular activity after physical exertion in horses [32,33]. Soroko et al. suggested that modifications in thermal distribution patterns are associated with increased muscular activity and enhanced regional blood perfusion occurring during physical exercise in horses [34]. Qualitative assessment of the thermographic images obtained in the present study revealed consistent alterations in thermal distribution patterns.

The present study also demonstrated the significant influence of physical exercise on the increase in body surface temperature within all evaluated regions of interest. Previous studies have similarly reported evident temperature increases following exercise, particularly in the shoulder region [35,36]. The differences observed between the mean surface temperatures of the forelimb and gluteal regions after training may reflect the distinct functional involvement of these muscle groups during locomotion [37,38,39,40]. The higher temperatures identified in the forelimb region could be associated with the greater biomechanical load supported by the thoracic limbs compared to the pelvic limbs during exercise in horses [41,42,43].

The relationship between rectal temperature and maximum local surface temperature differed between the two evaluated regions. In the BX1 region, the significant change in regression slope observed before and after exercise suggests that treadmill exercise modified the association between core thermoregulatory responses and localized muscular heat production [30]. Before exercise, rectal temperature was not positively associated with maximum local surface temperature, whereas after exercise, horses with higher rectal temperatures tended to exhibit higher maximum temperatures within the BX1 region. This finding likely reflects the physiological increase in muscle metabolism and peripheral blood flow during exercise, resulting in greater heat transfer to the skin surface [18,19]. In contrast, the relationship between rectal temperature and maximum local surface temperature remained unchanged in the EL1 region. The absence of a significant difference between regression slopes suggests that the thermal response of the gluteal musculature may be influenced by additional physiological factors beyond changes in rectal temperature, including regional muscle recruitment, local perfusion, and individual variation in heat dissipation during exercise [13,18].

Previous studies by Uddin et al. [44,45] demonstrated the superiority of maximum temperature analysis over mean temperature for detecting subtle thermal changes in animals, providing a detailed comparison between mean and maximum infrared thermography (IRT) measurements. The inclusion of maximum local surface temperature analysis provided complementary information to mean temperature measurements by highlighting focal thermal responses within the ROI [46]. Previous studies have suggested that peak temperature is particularly useful for identifying localized increases in metabolic activity, inflammation, and musculoskeletal stress, which may not be fully reflected by mean temperature values [46]. In contrast, mean local surface temperature provides information on the overall thermal response of the entire ROI and reflects more generalized physiological changes [47]. Therefore, both parameters were evaluated in the present study to obtain complementary information regarding exercise-induced thermal responses. In agreement with previous reports, both mean and maximum local surface temperatures increased significantly following treadmill exercise, confirming that each parameter contributes valuable information for the assessment of muscular activity in horses [46,47,48].

Mild sweating was occasionally observed after exercise, particularly in the cervical and shoulder regions; however, it was limited in extent and did not appear to interfere with the thermographic assessment of the Bx1 and El1 regions. Sweating represents an important thermoregulatory response to exercise in horses, facilitating heat dissipation through evaporative cooling and potentially influencing skin surface temperature during and after physical effort [48,49]. Thermographic measurements are also highly susceptible to external factors, particularly ambient temperature and hair coat characteristics, which may influence the recorded surface temperature [50]. Previous studies have demonstrated that ambient temperature and variations in hair coat length should be considered when interpreting thermographic measurements in horses [51]. In the present study, these potential sources of variability were minimized by maintaining relatively stable ambient temperature throughout the experimental period and by standardizing the hair length within the evaluated regions before each measurement session. Together with the standardized imaging protocol, these conditions likely minimized the influence of external factors on the recorded thermal responses [51]. Similar observations regarding thermographic assessment under controlled exercise conditions have been reported previously [47,50].

A limitation of the present study is the relatively small number of horses included in the experimental protocol. Although repeated measurements increased the total number of observations and the mixed-effects model accounted for within-subject dependence, the findings should be considered preliminary.

The results of the present study demonstrated a significant increase in surface temperature within the Bx1 and El1 regions following exercise, accompanied by visible modifications in thermal distribution patterns. Future studies including larger and more diverse cohorts of horses are warranted to validate these findings and establish their broader applicability. In addition, further investigations should: (1) assess temperature changes following different exercise intensities and durations; (2) compare thermographic responses among horses of different breeds, ages, and training levels; (3) compare thermographic responses in horses trained during the morning and afternoon to evaluate the influence of training time on surface temperature patterns; and (4) determine the potential value of infrared thermography as a tool for monitoring training adaptation and early detection of musculoskeletal abnormalities in sport horses.

## 5. Conclusions

The present study demonstrated significant increases in surface temperature within the Bx1 and El1 regions following exercise, accompanied by visible changes in thermal distribution patterns. The results did not support the null hypothesis, as significant differences were identified between thermographic evaluations performed before and after training. These findings indicate that infrared thermography is capable of detecting physiological thermal changes associated with muscular activity and exercise in horses.

Nevertheless, these findings should be considered preliminary because of the limited number of horses included in the study. Further studies involving larger and more diverse equine populations are needed to validate these results, confirm their broader clinical applicability, and establish the role of infrared thermography as an objective tool for monitoring exercise-induced thermal responses in horses.

## Figures and Tables

**Table 1:** Daily ambient temperature, relative humidity, and air movement recorded during treadmill training and thermographic assessment.

Study Day	Ambient Temperature (°C)	Relative Humidity (%)
1	21.3	72
2	21.4	71
3	21.6	73
4	21.4	70
5	21.6	73
6	21.2	72
7	21.6	74
8	21.5	72
9	21.4	73
10	21.7	75

**Table 2:** Rectal temperature (°C) recorded before (T1) and immediately after (T2) treadmill exercise in horses.

Study day	Horse A		Horse B		Horse C	
	T1 (°C)	T2 (°C)	T1 (°C)	T2 (°C)	T1 (°C)	T2 (°C)
1	37.1	38.4	37.3	38.2	37.2	38.6
2	37.5	38.6	37.4	38.7	37.6	38.9
3	37.6	38.5	37.5	38.2	37.3	38.3
4	38.7	38.7	37.8	38.6	37.9	39.4
5	37.8	38.2	37.2	38.1	37.3	38.2
6	37.5	39.1	37.8	38.5	37.6	38.4
7	37.4	38.8	37.9	38.3	37.6	38.6
8	37.2	39.4	37.3	39	37.4	38.6
9	37.7	39.1	37.4	39.2	37.5	38.2
10	37.2	38.7	37.6	38.8	37.5	38.6

**Table 3:** Individual mean local surface temperatures (°C) recorded in the BX1 region before (T1) and immediately after (T2) treadmill exercise.

Analysis Variable: Local Average Temperature						
Evaluation Moment	N Obs	Mean	Std Dev	Minimum	Maximum	N
Before training	30	33.75	0.21	33.40	34.20	30
After training	30	35.36 ***	0.16	35.10	35.80	30
*p*-value (after -before)	<0.001					

*** Very high statistically significant differences between before and after training temperatures.

**Table 4:** Individual mean local surface temperatures (°C) recorded in the EL1 region before (T1) and immediately after (T2) treadmill exercise.

Analysis Variable: Local Temperature						
Evaluation Moment	N Obs	Mean	Std Dev	Minimum	Maximum	N
Before training	30	32.37	0.18	32.00	32.80	30
After training	30	33.33 ***	0.24	32.80	33.80	30
*p*-value (after—before)	<0.001					

*** Very high statistically significant differences between before and after training temperatures.

**Table 5:** Descriptive statistics of maximum local surface temperatures recorded in the BX1 and EL1 regions and rectal temperature before and immediately after treadmill exercise.

Moment	N	Variable	Mean	Std Dev	Std Error	Lower 95% CL for Mean	Upper 95% CL for Mean
Before training	30	Rectal temperature	37.53	0.31	0.06	37.41	37.64
		Bx1 maximum temperature	33.60	0.64	0.12	33.36	33.84
		El1 maximum temperature	34.56	0.23	0.04	34.47	34.64
After training	30	Rectal temperature	38.63	0.36	0.07	38.49	38.77
		Bx1 maximum temperature	34.55	0.30	0.06	34.43	34.66
		El1 maximum temperature	35.90	0.18	0.03	35.83	35.97

## Data Availability

The data supporting the findings of this study are available within the article and its Supplementary Materials. Additional information may be obtained from the corresponding author upon request.

## Supplementary Materials

The following supporting information can be downloaded at: https://www.mdpi.com/article/10.3390/ani16162517/s1, Consent for Cooperation; Etic and project approval; Table S1: Daily mean and maximum BX1 temperatures before (T1) and after (T2) treadmill exercise; Table S2: Daily mean and maximum El1 temperatures before (T1) and after (T2) treadmill exercise; Video S1: Training on treadmill at a speed of 2 m per second.
