# Supplementary material for: Infrared Thermographic Assessment of Exercise-Induced Changes in Local Surface Temperature in Horses Following Treadmill Training: Preliminary Findings
Source: Animals (Basel). 2026 Aug 12;16(16):2517. doi: 10.3390/ani16162517 (PMC13508984; doi:10.3390/ani16162517)
Supplement: Supplementary file 1 [file animals-16-02517-s001.zip › Table S1 - Daily mean and maximum BX1 temperatures before (T1) and after (T2) treadmill exercise.pdf]

Daily mean and maximum local surface temperatures (°C) recorded in the BX1 region before (T1) and immediately after (T2) treadmill exercise during the 10-day study period.

Study day	Bx1			
	Mean temperature		Maximum temperature	
	T1 (Mean, °C)	T2 (Mean, °C)	T1 (Mean, °C)	T2 (Mean, °C)
1	33.83	35.43	34.7	35.83
2	33.73	35.5	34.43	35.83
3	34.03	35.33	34.63	35.83
4	33.63	35.66	34.36	36.23
5	33.63	35.33	34.7	35.83
6	33.5	35.23	34.36	35.73
7	33.8	35.23	34.63	35.86
8	33.63	35.2	34.33	35.8
9	33.7	35.36	34.53	35.93
10	34.06	35.3	34.86	36.1
