# Supplementary material for: Infrared Thermographic Assessment of Exercise-Induced Changes in Local Surface Temperature in Horses Following Treadmill Training: Preliminary Findings
Source: Animals (Basel). 2026 Aug 12;16(16):2517. doi: 10.3390/ani16162517 (PMC13508984; doi:10.3390/ani16162517)
Supplement: Supplementary file 1 [file animals-16-02517-s001.zip › Table S2 - Daily mean and maximum El1 temperatures before (T1) and after (T2) treadmill exercise.pdf]

Daily mean and maximum local surface temperatures (°C) recorded in the El11 region before (T1) and immediately after (T2) treadmill exercise during the 10-day study period.

Study day	El1			
	Mean temperature		Maximum temperature	
	T1 (Mean, °C)	T2 (Mean, °C)	T1 (Mean, °C)	T2 (Mean, °C)
1	32.56	33.46	33.46	34.76
2	32.36	33.36	33.7	34.33
3	32.5	33.5	33.63	34.7
4	32.43	33.1	33.36	34.63
5	32.23	33.23	33.83	34.16
6	32.26	33.33	33.6	34.53
7	32.43	33.33	33.7	34.23
8	32.36	33.56	33.53	34.73
9	32.23	33.23	33.6	34.53
10	32.3	33.16	33.53	34.76
